# Protein conformation in a vaccine matters

**DOI:** 10.18632/oncotarget.5102

**Published:** 2015-08-05

**Authors:** Trudy G. Morrison

**Affiliations:** University of Massachusetts Medical School, Worcester, MA, USA

**Keywords:** Immunology and Microbiology Section, Immune response, Immunity, protein conformation, respiratory syncytial virus, vaccine, fusion protein

Vaccination is one of the most successful public health measures for control of viral diseases. Indeed, vaccines have either eliminated or considerably reduced many viral diseases worldwide. However, there are still many serious viral pathogens for which no vaccine is available. One of these pathogens is respiratory syncytial virus (RSV).

RSV is a significant threat to human health. The virus is the principle cause of acute viral lower respiratory tract infections in infants and young children. RSV results in, world wide, 38 million infections per year in children under 5 years of age with ten percent of those requiring hospitalization and up to 200,000 deaths per year [1]. Severe RSV infections during infancy have been correlated with the development of asthma in later years. In addition, the elderly, immunocompromised populations, stem cell transplant recipients, and individuals with cardiopulmonary diseases are also at risk for serious, potentially life threatening RSV disease [2].

RSV, a paramyxovirus, is an enveloped, negative-stranded RNA virus. While virions contain three surface glycoproteins, G, SH, and F proteins, it is the F protein that is considered the antigen that stimulates the most potent and broadest spectrum of protective antibodies. The paramyxovirus F protein mediates fusion of viral and cell membranes important for virus entry into cells. As with all paramyxovirus F proteins, the fusion competent molecule is folded and assembled into virus in a metastable, pre-fusion conformation. Upon activation of fusion during the onset of infection, the pre-fusion F protein undergoes major conformational changes refolding into a biologically inactive post fusion form and in the process mediating the post-fusion of viral and cellular membranes.

RSV vaccine development has been ongoing for decades without resulting in a licensed vaccine. Previously tested vaccine candidates have failed in human trials due to their failure to stimulate adequate levels of protective immunity. It has recently become clear that many of these vaccine candidates have likely failed because they contained primarily the post-fusion form of the F protein. Recent major advances from several laboratories hold promise to accelerate development of a vaccine. Most importantly, McLellan, et al solved the structure of the particularly unstable pre-fusion RSV F protein, revealed a novel antigenic site on the pre-fusion F protein, and, furthermore, identified mutations that stabilized the pre-fusion F protein [3, 4]. While the post-fusion F protein can stimulate neutralizing antibodies, these antibodies require much higher concentrations to neutralize virus than antibodies specific for the novel, pre-fusion F protein antigenic site, likely accounting for the failure of previously tested vaccine candidates.

We have recently reported incorporation of these stabilizing mutational changes into the RSV F protein associated with a virus-like particle (VLP) vaccine candidate and demonstrated a significantly improved protective response to this vaccine candidate in a murine system [5]. We have been developing VLPs as vaccine candidates for RSV with the goal of eliminating many of the problems that have been associated with more classical approaches to RSV vaccine development. VLPs are virus-sized particles released from cells expressing the major structural proteins of a virus but in the absence of a viral genome. VLPs, therefore, do not contain a genome and are thus noninfectious and likely safer than infectious, attenuated viruses or live virus vectored vaccine candidates. Conformation of proteins in VLPs can also be more easily manipulated than virion associated proteins since cell entry functions need not be maintained. Immune responses to an antigen are considerably improved if the antigen is associated with a virus-sized particle in an ordered, repetitive array [6]. VLP associated glycoproteins are expressed in an ordered array and retain authentic epitopes since they do not require chemical treatment with the accompanying conformational changes necessary for inactivated virus vaccine preparation. It has also been found that VLPs stimulate robust humoral and cellular immune responses without adjuvant addition, a complicating requirement for soluble protein vaccine candidates.

We have previously shown that VLPs containing the wild type RSV F protein stimulated durable, neutralizing antibody responses, protection from RSV challenge, and memory B cell responses in a murine system, in contrast to RSV infection [5]. Furthermore, we showed that induction of B cell memory depended upon the conformation of the F protein [7]. However, the neutralizing antibody titers induced by these VLPs were not as high as that deemed necessary for protection in humans although titers were much higher than those induced by RSV infection. While the wild type F protein in VLPs contained the pre-fusion specific epitope [7], the instability of the wild type F pre-fusion form likely resulted in the conversion of the pre-fusion to the post-fusion form during immunization. However, we found that incorporation of the stabilized pre-fusion F protein into VLPs was able to increase the neutralizing antibody titers, after a single injection of VLPs, by 5–6 fold compared to titers induced by wild type F containing VLPs and to levels well above the minimal titers necessary for protective antibodies [5].

VLPs are increasingly being considered as vaccine candidates for a variety of human and animal pathogens. Our results demonstrate that the conformation of antigens in VLPs is of critical importance for optimal stimulation of protective as well as durable immune responses.
